# Minimally Invasive and Full Sternotomy Aortic Valve Replacements Lead to Comparable Long-Term Outcomes in Elderly Higher-Risk Patients: A Propensity-Matched Comparison †

**DOI:** 10.3390/jcdd11040112

**Published:** 2024-03-31

**Authors:** Jan Hlavicka, Larissa Gettwart, Julian Landgraf, Razan Salem, Florian Hecker, Enis Salihi, Arnaud Van Linden, Thomas Walther, Tomas Holubec

**Affiliations:** Department of Cardiovascular Surgery, University Hospital Frankfurt and Goethe University Frankfurt, 60596 Frankfurt/Main, Germany; jan.hlavicka@centrum.cz (J.H.); larissa@gettwart.de (L.G.); jlandgraf1@gmail.com (J.L.); razan.salem@gmail.com (R.S.); florian.hecker@herz-frankfurt.de (F.H.); enis.salihi99@gmail.com (E.S.); arnaud.vanlinden@herz-frankfurt.de (A.V.L.); thomas.walther@herz-frankfurt.de (T.W.)

**Keywords:** aortic valve replacement, minimally invasive surgery, full sternotomy, ministernotomy

## Abstract

Background: Minimally invasive aortic valve replacement (AVR) via upper ministernotomy (MiniAVR) is a standard alternative to full sternotomy access. Minimally invasive cardiac surgery has been proven to provide a number of benefits to patients. The aim of this study was to compare the short- and long-term outcomes after MiniAVR versus conventional AVR via full sternotomy (FS) using a biological prosthesis in an elderly higher-risk population. Methods: Between January 2006 and July 2009, 918 consecutive patients received AVR ± additional procedures with different prostheses at our center. Amongst them, 441 received isolated AVR using a biological prosthesis (median age of 74.5; range: 52–93 years; 50% females) and formed the study population (EuroSCORE II: 3.62 ± 5.5, range: 0.7–42). In total, 137 (31.1%) of the operations were carried out through FS, and 304 (68.9%) were carried out via MiniAVR. Follow-up was complete in 96% of the cases (median of 7.6 years, 6610 patient-years). Propensity score matching (PSM) resulted in two groups of 68 patients with very similar baseline profiles. The primary endpoints were long-term survival, freedom from reoperation, and endocarditis, and the secondary endpoints were early major adverse cardiac and cerebrovascular events (MACCEs). Results: FS led to shorter cardio-pulmonary bypass and aortic cross-clamp durations: 90 (47–194) vs. 100 (46–246) min (*p* = 0.039) and 57 (33–156) vs. 69 (32–118) min (*p* = 0.006), respectively. Perioperative stroke occurred in three patients (4.4%; FS) vs. one patient (1.5%; MiniAVR) (*p* = 0.506). The 30-day mortality was similar in both groups (2.9%, *p* = 1.000). Survival at 1, 5, and 10 years was 94.1 ± 3% (FS and MiniAVR), 80.3 ± 5% vs. 75.7 ± 5%, and 45.3 ± 6% vs. 43.8 ± 6%, respectively (*p* = 0.767). There were two (2.9%) reoperations in each group and two thrombo-embolic events (2.9%) vs. one (1.5%) thrombo-embolic event in the MiniAVR and FS groups, respectively (*p* = 0.596). Conclusions: In comparison to FS, MiniAVR provided similar short- and long-term outcomes in a higher-risk elderly population receiving biological prostheses. In particular, long-term survival, freedom from reoperation, and the incidence of endocarditis were comparable. These results clearly advocate for the routine use of MiniAVR as a standard procedure for AVR, even in a high-risk population.

## 1. Introduction

Due to the increasingly high life expectancy, aortic stenosis has become one of the most common heart diseases. There are approximately 55 million patients older than 65 years with any form of aortic valve stenosis worldwide, and the number of patients is expected to increase to 75 million in 2030 [1]. The overall risk associated with conventional surgery using cardio-pulmonary bypass (CPB) and full sternotomy (FS) among elderly patients led to the search for alternative approaches [2], including transcatheter aortic valve implantation and aortic valve replacement (AVR) through minimally invasive approaches, for example, upper ministernotomy (MiniAVR). MiniAVR has proven to be effective and safe and has less complications postoperatively with comparable mid- and long-term mortality compared to full sternotomy (FS) [3,4,5,6]. Despite the limited access to the retrosternal space, the remaining sternal integrity and better quality of life with less pain [7] resulted in the request to perform AVR using a minimally invasive technique.

Despite the high potential of MiniAVR, which even allows concomitant procedures to be performed, some authors are hesitant to implement MiniAVR. This is mainly based on the longer learning curve and the longer cross-clamp and CPB times, potentially resulting in a worsening of renal and neurological functions [8,9]. In addition, most of the published papers do not take into consideration the current trend of surgery in high-risk patients, whose long-term prognosis could be improved fundamentally by a minimally invasive approach [10].

The aim of this study was to analyze the short- and long-term results of MiniAVR in comparison to FS in higher-risk patients with regard to long-term survival, freedom from reoperation, prosthetic valve endocarditis (PVE), and early major adverse cardiac and cerebrovascular events (MACCEs).

## 2. Materials and Methods

### 2.1. Ethics Statement

This study was conducted in accordance with the Declaration of Helsinki (as revised in 2013). This study was approved by the local Ethic Committee of University Hospital Frankfurt (No. 79/13, 13 May 2020), and written informed consent was waived due to the retrospective nature of this study. The following article is published in accordance with the STROBE reporting checklist.

### 2.2. Study Design and Patients’ Characteristics

**Figure 1:**
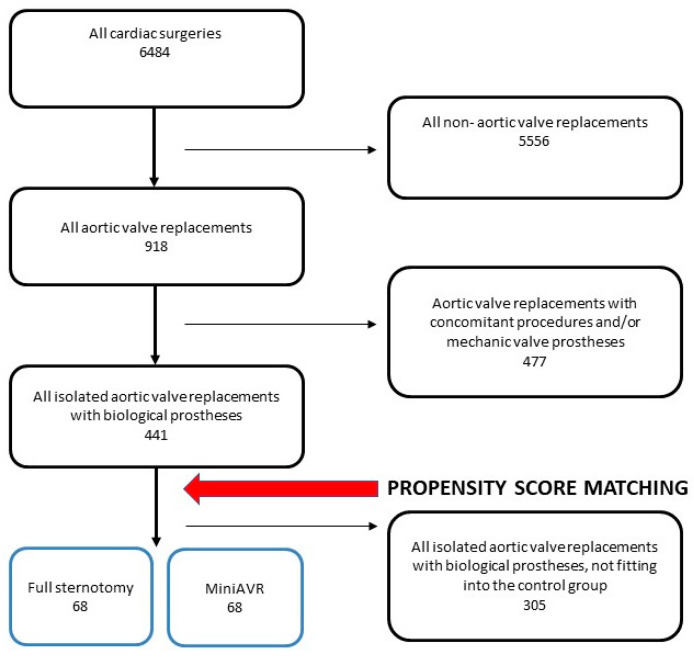
Consort-type diagram of patient selection for this study. MiniAVR—aortic valve replacement via upper ministernotomy.

Between January 2006 and July 2009, 918 patients underwent AVR at the University Hospital Frankfurt, Germany. Four hundred forty-one patients receiving an isolated AVR with a biologic prosthesis (median age of 74.5, range: 50–93 years, 50% females) were included in this single-center study. In total, 137 (31.1%) of the operations were carried out through FS, and 304 (68.9%) were carried out via MiniAVR. The exclusion criteria were any concomitant heart and aortic procedures, such as valve procedures and coronary artery bypass grafting, with the exception of surgery on the supracoronary ascending aorta and subaortic septal myectomy. The process of patient selection is depicted in Figure 1.

**Figure 2:**
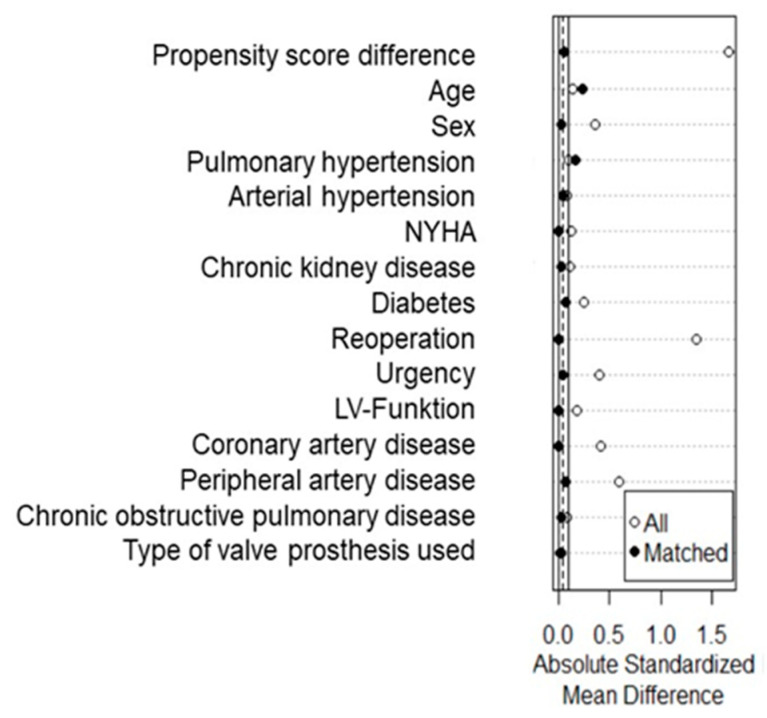
Absolute standardized mean differences of the variables used for propensity score matching. LV—left ventricle, NYHA—New York Heart Association functional class.

Propensity score matching was performed based on patient characteristics (age; sex; pulmonary hypertension; arterial hypertension; dyspnea according to the New York Heart Association functional class; chronic kidney disease; diabetes mellitus; reoperation; the urgency of the surgery; the ejection fraction of the left ventricle; the presence of coronary artery disease, peripheral artery disease, and chronic obstructive pulmonary disease; and type of valve prosthesis used), resulting in two groups of 68 receiving FS or MiniAVR (Figure 1 and Figure 2). These two groups were used for further comparison. The primary endpoints were defined as long-term survival, freedom from structural valve deterioration resulting in reoperation, thrombo-embolic events, and PVE at long-term follow-up. Early major adverse cardiovascular and cerebral events were defined as secondary endpoints.

### 2.3. Patient Management

All patients received preoperative coronary angiography as well as transthoracic echocardiography (TTE). Potential clinically relevant carotid artery sclerosis was excluded using Doppler ultrasound, and a lung function test was performed prior to surgery. Intraoperative transesophageal echocardiography was performed for all patients to evaluate prosthetic valve function and to assess other cardiac valves and left ventricular function during weaning from CPB. In the early postoperative phase, anticoagulation prophylaxis with subcutaneous low-molecular-weight heparin was administered 6 h after surgery. Oral antiplatelet therapy with Aspirin was administered from the first postoperative day until 3 months postoperatively. Only in patients with repeated postoperative atrial fibrillation was oral anticoagulation with coumadin initiated. All patients received TTE at discharge. All patients were prospectively followed by phone interviews and/or clinical assessment and TTE at our outpatient clinic. For patients not seen personally, we retrieved clinical assessment and echocardiography reports from the attending cardiologists. The follow-up of the unmatched cohort was completed in 96% of cases (median of 7.6 years for a total of 6610 patient-years). The median follow-up in the FS group was 7.5 years (range: 0–15) vs. 8.0 years (range: 0–15) in the MiniAVR group. Follow-up was completed in 98.5% of cases (two patients lost) in the matched cohort for a total of 1025 patient-years.

### 2.4. Surgical Technique

Among the patients whose operations used the minimally invasive technique, a J-shaped upper ministernotomy with extension into the 3rd or 4th intercostal space was performed. For better exposition based on the anatomic circumstances, arterial cannulation was performed either through the right subclavian artery (Fem Flex™, Edwards Lifesciences Corp., Irvine, CA, USA) or the distal aortic arch. An oval double-stage cannula (VC2TM; Medtronic, Inc., Minneapolis, MN, USA) was used for the venous cannulation of the right atrial appendage. The decompression and de-airing of the heart was performed with the placement of a ventricular vent through the right upper pulmonary vein. After cross-clamping and the administration of an antegrade non-selective cold blood cardioplegic solution (Calafiore; repeated every 20 min) to the ascending aorta, an oblique aortotomy was performed. After sizing the aortic annulus, the biologic prosthesis was implanted with a series of interrupted pledgeted and braided 2-0 sutures. Three types of stented bioprostheses were implanted: CE Perimount^®^ (Model 2900), CE Perimount Magna^®^ (Model 3000) (both Edwards Lifesciences Corp., Irvine, CA, USA), and Mosaic^®^ (Medtronic, Dublin, Ireland). In all cases, surgery was performed using mild hypothermia of 34 °C.

### 2.5. Statistical Analysis

All available data were collected retrospectively and entered into a Microsoft Excel, Version 2021 for Windows^®^ (Microsoft Corp., Redmond, WA, USA) spreadsheet. A Kolmogorov–Smirnov test was used to assess the normality of the data. Continuous and discrete variables were reported as means ± standard deviations or medians and ranges for data that were not normally distributed. Categorical and ordinal variables were reported using the numbers and percentages of observations. Continuous and discrete variables were compared using Kruskal–Wallis or Mann–Whitney tests, where appropriate. Categorical and ordinal variables were compared using Pearson’s Chi-squared test or Fischer’s exact test, where appropriate. A univariate and multivariate logistic regression analysis of predictors of mortality was performed to evaluate the associations between independent risk factors, comorbidities, and mortality. A multivariate Cox regression identified the independent risk factors of long-term mortality after AVR. The probabilities of survival and freedom from adverse events were calculated according to the Kaplan–Meier method. The survival and freedom-from-event curves were compared by a log-rank test. A two-sided *p*-value lower than 0.05 was defined as indicating statistical significance. Statistical analysis was performed using the IBM^®^ SPSS^®^ Statistics software program (version 29.0.0.0 for MS Windows, IBM Corporation, Armonk, NY, USA).

## 3. Results

OVERALL COHORT

### 3.1. Patients’ Characteristics

The demographics are presented in Table 1. Before matching, women were represented less often in the FS group (41.6 vs. 53.3%; *p* = 0.024). In comparison with the FS group, the MiniAVR group had fewer previous cardiac surgeries (20.4% vs. 1.6%; *p* < 0.001); a lower rate of moderate or severe left ventricular dysfunction (23.0% vs. 14.6%; *p* = 0.039); a better ejection fraction (55 ± 13% vs. 59 ± 12%; *p* = 0.042); and fewer comorbidities such a coronary artery disease (36.5% vs. 18.8%; *p* < 0.001), diabetes (34.0% vs. 23.7%; *p* = 0.034), preoperative stroke (11.9% vs. 3.7%; *p* = 0.002), and ischemic limb disease (12.8% vs. 5.2%; *p* = 0.013). The incidence of moderate pulmonary hypertension was slightly higher in the MiniAVR group; however, this was not statistically significant (*p* = 0.091). These differences all disappeared after propensity score matching (Figure 2).

### 3.2. Operative Data

The operative data are shown in Table 2. We observed a significantly shorter aortic cross-clamp time in the FS group with a median of 59 min (33–190) vs. 70 min (29–131) in the MiniAVR group (*p* < 0.001). The CPB time, however, did not differ significantly: median of 93 min (47–307) in the FS group vs. 101 min (46–246) in the MiniAVR group (*p* = 0.075). There were no significant differences between the models of the valve prostheses used (*p* = 0.624).

### 3.3. Early Postoperative and Follow-Up Results

The early postoperative data of patients pre- and post-matching are shown in Table 3. Fifteen patients (4.9%) died in the MiniAVR group during the first 30 days, compared with seven (5.1%) cases in the FS group (*p* = 0.635). The other early postoperative complications, such a re-exploration for bleeding, stroke, renal insufficiency, AV block requiring pacemaker implantation, low cardiac output syndrome, and wound healing disorder, did not differ significantly between the groups. The 1-, 5-, and 10-year survival rates were 87.6 ± 2.8%, 73.1 ± 3.9%, and 42.2 ± 4.4% in the FS group vs. 87.8 ± 1.9%, 73.9 ± 2.6%, and 45.3 ± 2.9% in the MiniAVR group, respectively (*p* = 0.134). The incidence of reoperation during follow-up was very low: in total, 2.2% of patients in the FS group and 5.3% of the MiniAVR group (*p* = 0.205) had another operation due to prosthesis deterioration or infective PVE. The incidence of PVE was also very low in the long term (2.2% vs. 3.0%; *p* = 0.762). During the long-term follow-up, thrombo-embolic events occurred more frequently in the MiniAVR group: 1.5% of FS patients vs. 5.9% of MiniAVR patients (*p* = 0.049).

PROPENSITY SCORE-MATCHED COHORT

### 3.4. Patients’ Characteristics

The baseline characteristics of the matched cohort are provided in Table 1. No statistically significant differences were observed between the FS and MiniAVR groups.

#### Operative Data

The operative data after PSM are shown in Table 2. The patients in the FS group needed a shorter CPB time (90 (47–194) vs. 100 (46–246) min; *p* = 0.039), as well as a shorter cross-clamp time (57 (33–156) vs. 69 (32–118) min; *p* = 0.006).

### 3.5. Early Postoperative and Follow-Up Results

**Figure 3:**
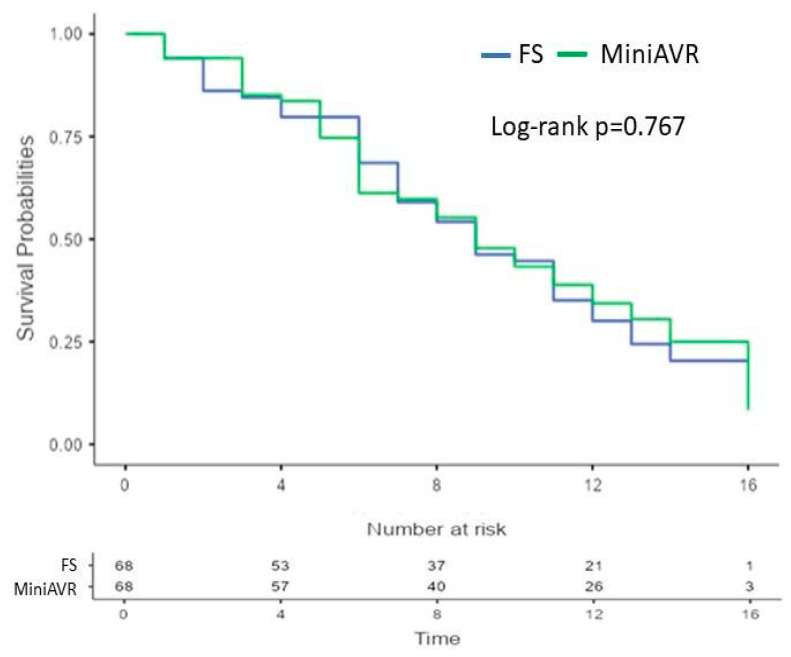
Kaplan–Meier curves showing survival after AVR post-PSM. Time expressed in years. AVR—aortic valve replacement, FS—full sternotomy, MiniAVR—aortic valve replacement via upper ministernotomy, PSM—propensity score matching.

**Figure 4:**
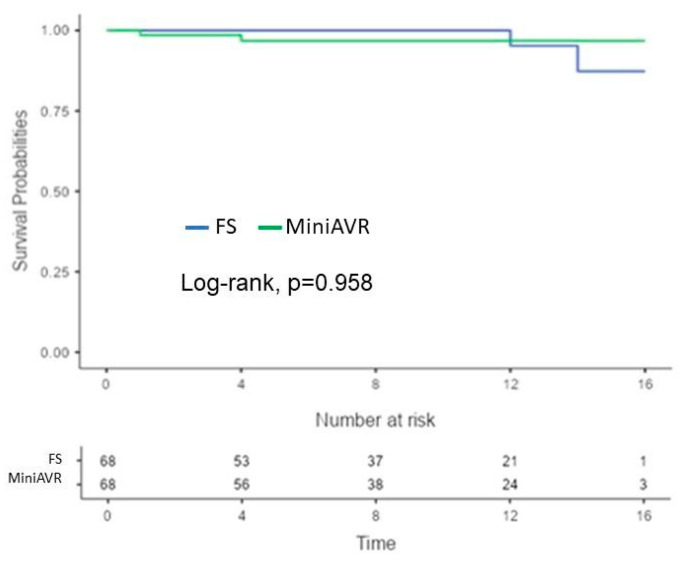
Kaplan–Meier curves showing freedom from aortic valve-related reoperation after AVR post-PSM. Time expressed in years. AVR—aortic valve replacement, FS—full sternotomy, MiniAVR—aortic valve replacement via upper ministernotomy, PSM—propensity score matching.

The incidence of major early postoperative complications was low in both cohorts after PSM, as shown in Table 3. No patients died during their postoperative hospital stays, and two patients died (2.9%) in each group within 30 days postoperatively. The larger wound area in patients with a full sternotomy was not associated with a higher need for early re-exploration because of bleeding or heart tamponade (8.8% vs. 4.4%; *p* = 0.493). According to the Kaplan–Meier survival analysis, there was no significant difference in long-term mortality (Figure 3). The 1-, 5-, and 10-year survival rates were 94.1 ± 3%, 80.3 ± 5%, and 45.3 ± 6% in the FS group vs. 94.1 ± 3%, 75.7 ± 5, and 43.8 ± 6 in the MiniAVR group, respectively (*p* = 0.767). After PSM, the reoperation rates due to thrombotic or degenerative valve disorder were equal in both groups (5.9% vs. 5.9%; *p* = 1.000). Most of these operations occurred more than ten years postoperatively (Figure 4).

**Figure 5:**
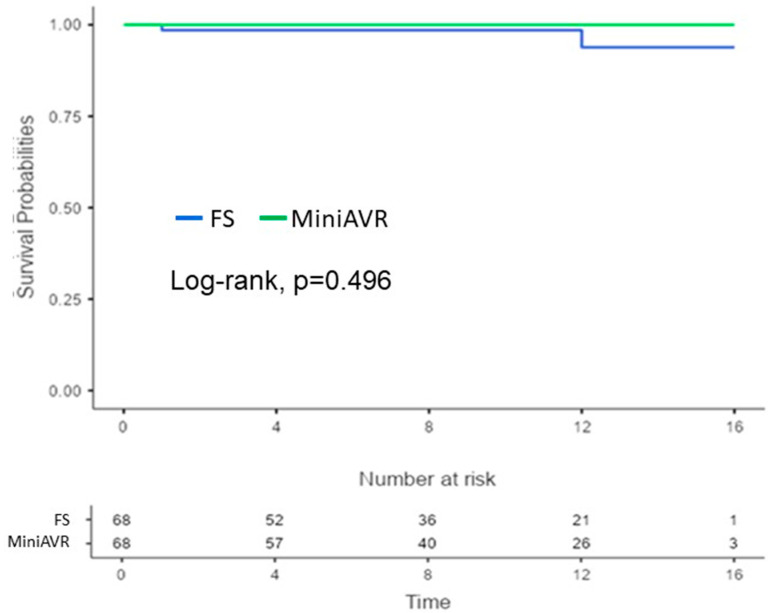
Kaplan–Meier curves showing freedom from aortic valve prosthetic endocarditis after AVR post-PSM. Time expressed in years. AVR—aortic valve replacement, FS—full sternotomy, MiniAVR—aortic valve replacement via upper ministernotomy, PSM—propensity score matching.

**Figure 6:**
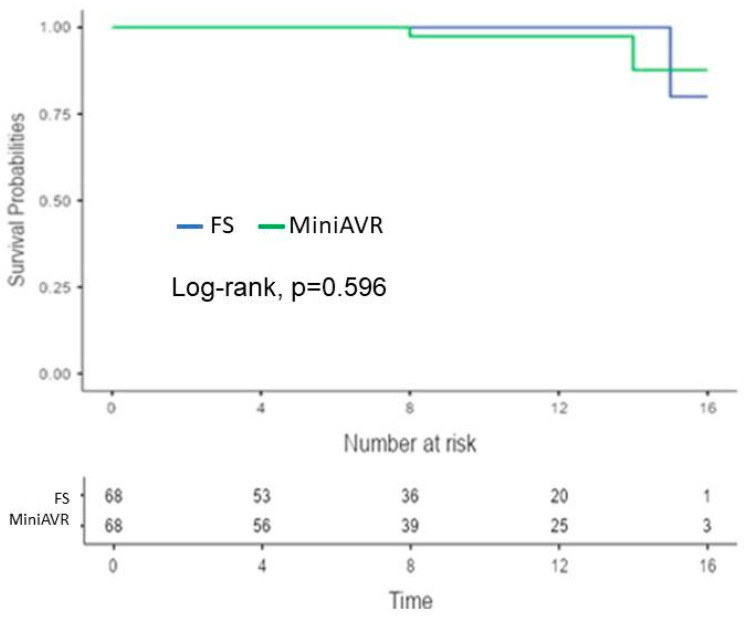
Kaplan–Meier curves showing freedom from thrombo-embolic events after AVR post-PSM. Time expressed in years. AVR—aortic valve replacement, FS—full sternotomy, MiniAVR—aortic valve replacement via upper ministernotomy, PSM—propensity score matching.

Prosthetic valve endocarditis was observed in two cases in the FS group after 12 years, while no endocarditis was observed in the MiniAVR group (2.9% vs. 0%; *p* = 0.496) (Figure 5). Thrombo-embolic events proved to be rare complications during the follow-up. Two patients in the MiniAVR group (2.9%) and one patient in the FS group (1.5%) experienced thrombo-embolic events after more than 8 years (*p* = 0.596; Figure 6).

## 4. Discussion

In our single-center study, we aimed to compare, by means of PSM analysis, the short- and long-term results of a higher-risk elderly population after AVR using a biological prosthesis via conventional full sternotomy or ministernotomy. The PSM analysis was able to eliminate confounding factors successfully, as both cohorts (FS and MiniAVR) were also very similar in terms of the prosthesis models used, valve cuspidity, pathology, and the urgency of the surgery.

Although the preoperative EuroSCORE II is formally considered a medium operative risk, the age (74.5 ± 7.0 (FS) vs. 76.15 ± 7.0 (MiniAVR)) of the patients places them within the higher-risk group. The advanced age per se is an independent risk factor for a prolonged length of stay, as Sharony at al. demonstrated in their multivariate regression analysis of a similar group of patients [9]. Also, Kaneko et al. substantiated higher age as a significant predictive factor in the decreased postoperative survival among reoperated octogenarians (HR, 1.15; 95% CI, 1.05–1.26; *p* = 0.002) [11]. The cross-clamp time and CPB time were significantly shorter in the FS group, which was in line with previously published studies [3,12]. Although minimally invasive AVR was a standard technique in our department at the time of this study, surgery with limited access is technically more demanding and requires careful patient selection, planning, and surgical technique. That is why the very first patients receiving MiniAVR during the learning curve of the surgeons were not included in this study. However, as the authors of the following studies as well as a pooled analysis of propensity-matched data with similar results pointed out, a longer mean cross-clamp time of 8–10 min (CI: 4.2554–14.59) and a longer mean CPB time of 8–11 min (CI: 1.42–21.06) did not lead to any clinically relevant consequences [5,6,13]. One of the largest propensity score comparisons between FS, MiniAVR, and right anterior minithoracotomy published by Mikus et al., comparing three groups of 377 patients, showed significantly reduced CPB and cross-clamp times in the latter group. Similar to our conclusions, the authors stated that minimally invasive AVR offers comparable short-term outcomes to FS despite a longer learning curve. Renal failure (odds ratio, 5.4; 95% confidence interval, 2.3 to 11.4; *p* < 0.0001), extracardiac arteriopathy (odds ratio, 2.9; 95% confidence interval, 1.1 to 6.7; *p* = 0.017), and the left ventricular ejection fraction (odds ratio, 0.96; 95% confidence interval, 0.93 to 0.99; *p* = 0.009) emerged as independent predictors of in-hospital mortality [14]. Only the EuroSCORE (OR, 1.52; 95% CI, 1.12–2.06; *p* < 0.01) was identified as an independent predictor of intrahospital mortality in the propensity-matched population comparing isolated FS and minithoracotomy [15].

In concordance with a retrospective propensity-matched analysis by Shehada et al. with very low 30-day mortality rates of 1.5 vs. 1.7% (*p* = 0.74) in the MiniAVR and FS groups [16], we also did not observe a significant difference between the groups in our study (both 2.95%; *p* = 1.000). An explanation for the higher 30-day mortality in our study is definitely that our patients were more than 10 years older compared to those in the mentioned study (cohort studied by Shehada et al.: 65.0 ± 10.5 vs. 65.7 ± 11.5 years, *p* = 0.230; our cohort: 74.5 ± 7.0 vs. 76.15 ± 7.0 years, *p* = 0.212). Better short-term survival favoring MiniAVR, as demonstrated in a comparable study published by Merk et al., could not be confirmed in our study. Their in-hospital mortality rates of 0.4 vs. 2.3% (*p* = 0.013) were higher than ours (no patients died in our study before discharge) [17]. Nevertheless, the 2.9% mortality in both matched groups was considerably low with respect to the EuroSCORE II values of 3.5 and 3.6, respectively. The low influence of the surgical technique (MiniAVR vs. FS) on early mortality was also confirmed by Lim et al. [13]. It is necessary to underline that our patients underwent surgical treatment between 2006 and 2009. Advanced techniques for surgical valve implantation and the use of sutureless valves, automatic suture devices, and low-profile cannulas are expected to shorten surgical times, possibly resulting in better postoperative outcomes [18].

Our study did not confirm the finding of Gilmanov et al. that MiniAVR significantly reduces ventilation time (median of 8 vs. 7 h (*p* = 0.022) in their study compared to 12 vs. 10 h (*p* = 0.204) in our study). However, there was no impact on in-hospital mortality (both groups were 1.64% in their study vs. 0% in both groups in our study) [19]. Importantly, their retrospective analysis of the minimally invasive group not only included upper ministernotomy but also right anterior thoracotomy. Such an inhomogeneous set of patients is difficult to compare with any control group. Welp et al. proved a significant reduction in ventilation time among obese patients whose operations were carried out through upper ministernotomy (8 vs. 6 h; *p* = 0.004). The benefit of minimally invasive access in their patients with BMI values over 32 was certainly greater than in our group of only slightly overweight patients (BMI 28) [20].

Long-term survival rates after 4 and 8 years of 89.3 ± 2.4% and 77.7 ± 4.7% vs. 81.8 ± 2.2% and 72.8 ± 3.1%, respectively (*p* = 0.034), published by Merk et al. were comparable to our results of 94 ± 3%, 80 ± 5%, and 45 ± 6% (FS) vs. 94 ± 3%, 75 ± 5, and 43 ± 6 (MiniAVR), respectively (*p* = 0.767), after 1, 5, and 10 years (Figure 2). Here, the matched patients in our study were more than 8.4 years older in the MiniAVR group and more than 7 years older in the FS group [17]. The aging of the population is an important circumstance in contemporary cardiac surgery. Older age and its associated comorbidities negatively affect patient survival, regardless of the chosen surgical strategy.

In terms of the incidence of low cardiac output syndrome, our patients had very good results with a generally low incidence of this severe postoperative complication with one implantation of ECMO in the FS group (1.5%) and two implantations of IABP in the MiniAVR group (2.9%) (*p* = 0.261). These findings are in line with similar findings by Aliahmed et al. [5] and others [19,21]. Aortic valve insufficiency grades 3 and 4, mostly associated with postoperative LCO, were present in only 20.6 and 17.6% of cases, respectively.

The overall incidence of prosthetic valve endocarditis (PVE) was very low in our study: 5.2% at 10 years. Only two patients (2.9%) in the FS group were hospitalized due to PVE during the 12 years after surgery in our study. No PVE occurred in the MiniAVR group after 12 years, even though our patients presented several identified risk factors for PVE (diabetes 26.5 vs. 23.5%, atrial fibrillation 26.5 vs. 23.5%, and male sex 57.4 vs. 55.9%) [22]. In their observational, population-based cohort study, Glaser et al. included 26 580 patients operated on using isolated AVR. During the median follow-up of 6.2 years (max. 18 years), the incidence of PVE was 3.5% [23]. The better results in our study are most likely associated with the later enrollment in the study (2006–2009 vs. 1995–2012) and the use of newer and more modern valve prostheses. Glaser et al., as well as Brennan et al., also found biological valve prostheses to be an independent risk factor for PVE compared with mechanical prostheses (HR of 1.53 (CI: 1.25–1.86) and HR of 1.6 (CI: 1.31–1.94)) [23,24]. Our findings, with a generally very low incidence of PVE of the biological prosthesis, do not support this statement, which is in line with The European Society of Cardiology Guidelines for the Management of Infective Endocarditis. The lack of a statistically significant difference between the groups in our study also supports the postulation that the chosen surgical technique does not play an important role in the incidence of PVE in the short and long terms. There is generally a lack of data on the occurrence of serious complications after MiniAVR in the long term. Compared to the retrospective study of late cerebrovascular events after transcatheter AVR from Muntané-Carol et al. [25], with 5.1% of patients presenting with stroke among 3750 patients within 2 years after implantation (80.5% ischemic, 18.8% hemorrhagic, and 0.7% undetermined), only three cases of thrombo-embolic events and stroke occurred in both matched groups (4.4%) in our study in the hospital and during follow-up (median: 7.6 years). The low incidence of such a serious, disabling complication may serve as a justification for a surgical approach to aortic stenosis. An additional satisfactory outcome was a very low incidence of PVE during follow-up (2.2% vs. 3.0% (*p* = 0.762) in the overall cohort and 2.9% vs. 0% (*p* = 0.496) in the matched groups), which was consistent with the findings of Moriyama et al. (4333 surgical patients with bioprostheses, mean follow-up of 4.2 ± 2.6 years, PVE incidence of 1.2%) [26] and a meta-analysis by Ando et al. (1866 surgical patients, mean follow-up of 3.4 years, PVE incidence of 1.3%) [27]. However, our patients (74.5 ± 7.0 to 76.15 ± 7.0 years old) did not confirm advanced age as a predictor of PVE after surgical AVR, as identified by Grubitzsch et al. It is difficult to compare minimally invasive surgical approaches with TAVR, with incidences of PVE in the long term ranging from 0.7% (mean follow-up: 3.1 ± 1.7) [28] to 2.0% (mean follow-up: 3.4 years) [27], because of inconsistent data and incomparable predictors of PVE (i.e., groin access, crimping of valve leaflets, suboptimal sterility, paravalvular regurgitation vs. prolonged cardio-pulmonary bypass, sternal wound infection, urinary tract infection, and intravascular catheter infection) [29]. Because of the low incidence of MACCEs in the long term, surgical AVR remains comparable to TAVR.

TAVR currently remains the method of choice for the treatment of aortic valve stenosis among high-risk and older patients. The most cited PARTNER study showed that TAVR and surgical valve replacement have comparable outcomes, despite the high number of moderate to severe paravalvular regurgitations (7%) within 1 year, which require surgical revision and are associated with increased mortality [30]. Comparable results at 1 year are to be expected in patients that undergo additional operations through partial upper sternotomy because of degenerated aortic valves and in patients requesting valve-in-valve (VIV) TAVR. Survival is even better in patients treated surgically (92% vs. 85.8%), as Kaneko et al. demonstrated [11,31], despite the ongoing problem with high valve gradients and potential ostial coronary obstruction after VIV TAVR.

### Limitations and Strengths

This study provides long-term results (over 10 years) for patients with higher risk at an advanced age, which is one of the most discussed topics at present. The results of our study may contribute to a consensus on minimally invasive AVR.

This study has several limitations. This is a retrospective, single-center, observational study, and all inherent disadvantages apply. Additionally, the judgements, skills, and habits of the surgeon played an important role in choosing one technique over the other. Despite very good PSM, the relatively limited number of matched patients may cause difficulties in interpreting the data.

## 5. Conclusions

Our data support the current clinical view that minimally invasive AVR through upper ministernotomy is safe, reproducible, and effective among higher-risk older patients, without any negative impact on long-term survival. The minimally invasive approach, as well as the use of biological prostheses, does not increase the risk of late endocarditis and thrombo-embolic events.

## Figures and Tables

**Table 1:** Demographic data and baseline characteristics.

	Unmatched Groups			Propensity Score-Matched Groups		
Variables ^a^	FS (*n* = 137)	MiniAVR (*n* = 304)	*p*-Value	FS (*n* = 68)	MiniAVR (*n* = 68)	*p*-Value
Age	74.1 ± 7.5	74.8 ± 7.3	0.581	74.5 ± 7.0	76.15 ± 7.0	0.212
BMI	27.25 ± 4.5	27.1 ± 4.4	0.869	27.6 ± 4.0	28.0 ± 4.0	0.625
ES II	4.4 ± 5.5 (0.7–42)	3.3 ± 3.6 (0.7–36)	0.006	3.5 ± 3.4 (1–27)	3.6 ± 5.0 (1–36)	0.670
Male	80 (58.4)	142 (46.7)	0.024	37 (57.4)	38 (55.9)	1.000
NYHA > 2	72 (54.5)	184 (60.7)	0.245	35 (51.5)	35(51.5)	1.000
PH			0.303			0.091
Moderate	89 (65)	217 (71.4)		39 (57.4)	51 (75.0)	
Severe	9 (6.6)	21 (6.9)		6 (8.8)	3 (4.4)	
HR			0.424			0.843
SR	109 (79.6)	252 (82.9)		50 (73.5)	52 (76.5)	
AF	28 (20.4)	52 (17.1)		18 (26.5)	16 (23.5)	
Redo	28 (20.4)	5 (1.6)	<0.001	3 (4.4)	3 (4.4)	1.000
LV EF			0.077			0.849
>55%	81 (60)	218 (72.2)		41 (60.3)	45 (66.2)	
45–55%	23 (17)	40 (13.2)		15 (22.1)	11 (16.2)	
30–45%	27 (20)	39 (12.9)		11 (16.2)	11 (16.2)	
<30%	4 (3)	5 (1.7)		1 (1.5)	1 (1.5)	
CHD	50 (36.5)	57 (18,8)	<0.001	14 (20.6)	14 (20.6)	1.000
AH	109 (89.3)	247 (86.7)	0.516	60 (88.2)	61 (89.7)	1.000
DM	45 (34.1)	71 (23.7)	0.034	18 (26.5)	16 (23.5)	0.843
COPD	36 (29.8)	70 (25.1)	0.388	20 (29.4)	19 (27.9)	1.000
CKD	53 (38.7)	92 (30.3)	0.100	23 (33.8)	22 (32.4)	1.000
Stroke	16 (11.9)	11 (3.7)	0.002	7 (10.4)	4 (5.9)	0.122
PAD	16 (11.9)	15 (5.2)	0.013	4 (5.9)	5 (7.4)	1.000
AI > 2	33 (24.1)	54 (17.8)	0.154	14 (20.6)	12 (17.6)	0.828

^a^ Data are presented as mean ± SD, median (range), or number (%), where appropriate. AF—atrial fibrillation, AH—arterial hypertension, AI—aortic insufficiency, BMI—body mass index, CHD—coronary heart disease, CKD—chronic kidney disease, COPD—chronic obstructive pulmonary disease, DM—diabetes, ES II—EuroSCORE II, FS—full sternotomy, HR—heart rhythm, LV EF—left ventricle ejection fraction, MiniAVR—aortic valve replacement via upper ministernotomy, NYHA—New York Heart Association functional class, PAD—peripheral artery disease, PH—pulmonary hypertension, Redo—reoperation, SR—sinus rhythm.

**Table 2:** Operative data.

	Unmatched Groups			Propensity Score-Matched Groups		
Variables ^a^	FS (*n* = 137)	MiniAVR (*n* = 304)	*p*-Value	FS (*n* = 68)	MiniAVR (*n* = 68)	*p*-Value
Aortic valve anatomy			0.037			0.974
Tricuspid	115 (83.9)	240 (78.9)		56(82.4)	55 (80.9)	
Bicuspid	18 (13.1)	62 (20.4)		11(16.2)	12 (17.6)	
Prosthesis	4 (2.9)	2 (0.7)		1(1.5)	1 (1.5)	
Indication			<0.001			0.321
Stenosis	99 (72.3)	251 (82.6)		49(72.1)	56 (82.4)	
Insufficiency	18 (13.1)	11 (3.6)		8(11.8)	4 (5.9)	
Combination	20 (14.6)	42 (13.8)		11(16.2)	8 (11.8)	
Urgency			<0.001			0.976
Elective	97 (70.8)	259 (85.2)		55(80.9)	54 (79.4)	
Urgent	31 (22.%)	44 (14.5)		12(17.6)	13 (19.1)	
Emergency	9 (6.6)	1 (0.3)		1(1.5)	1 (1.5)	
Model of the valve			0.624			0.315
CE Perimount	115 (83.9)	253 (83.2)		57(83.8)	53 (77.9)	
CE Perimount Magna	1 (0.7)	6 (2.1)		0 (0)	2 (2.9)	
Medtronic Mosaic	21 (15.3)	45 (14.8)		11 (16.2)	13 (19.1)	
CPB time	93 (47–307)	101 (46–246)	0.075	90 (47–194)	100 (46–246)	0.039
Cross-clamp time	59 (33–190)	70 (29–131)	<0.001	57 (33–156)	69 (32–118)	0.006
Second bypass	2 (1.5)	12 (3.9)	0.243	1 (1.5)	4 (5.9)	0.366

^a^ Data are presented as median (range) or number (%). CE—Carpentier-Edwards, CPB—Cardio-pulmonary bypass, FS—full sternotomy, MiniAVR—aortic valve replacement via upper ministernotomy.

**Table 3:** Postoperative data and follow-up.

	Unmatched Groups			Propensity Score-Matched Groups		
Variables ^a^	FS (*n* = 137)	MiniAVR (*n* = 304)	*p*-Value	FS (*n* = 68)	MiniAVR (*n* = 68)	*p*-Value
Wound healing disorder	13 (9.5)	27 (8.9)	0.859	6 (8.8)	6 (8.8)	1.000
Re-exploration	14 (10.2)	29 (9.5)	0.863	6 (8.8)	3 (4.4)	0.493
Stroke			0.053			0.506
TIA	1(1.5)	0		1 (1.5)	0 (0)	
Major insult	3 (2.2)	1 (0.3)		2 (2.9)	1 (1.5)	
CVVHD	13 (9.5)	24 (7.9)	0.581	6 (8.8)	3 (4.4)	0.493
AV block	13 (9.5)	21 (6.9)	0.341	5 (7.4)	5 (7.4)	1.000
Permanent pacemaker	5 (3.6)	8 (2.6)	0.553	1 (1.5)	1 (1.5)	1.000
Low cardiac output			0.378			0.261
Medication	6 (4.4)	7 (2.3)		1 (1.5)	0 (0)	
IABP	0 (0)	3 (1.0)		0 (0)	2 (2.9)	
ECMO	1 (0.7)	1 (0.3)		1 (1.5)	0 (0)	
Early mortality			0.635			1.000
Intrahospital	0 (0)	2 (0.7)		0 (0)	0 (0)	
30-day	7 (5.1)	15 (4.9)		2 (2.9)	2 (2.9)	
ICU stay (days)	2 (0–25)	2 (0–51)	0.706	1 (1–17)	2 (1–44)	0.293
Ventilation time (h)	12 (5–842)	10 (3–1153)	0.012	12 (5–192)	10 (3–812)	0.204
EF at discharge (%)	54.6 ± 8.0	59.0 ± 8.8	0.157	54.0 ± 9.0	53.0 ± 9.0	0.872
Follow-up						
Embolic event	2 (1.5)	18 (5.9)	0.046	1 (1.5)	2 (2.9)	0.596
Endocarditis	3 (2.2)	9 (3.0)	0.762	2 (2.9)	0 (0)	0.496
Reoperation	3 (2.2)	16 (5.3)	0.205	2 (2.9)	2 (2.9)	1.000
Complication + Reoperation	5 (3.6)	25 (8.2)	0.101	4 (5.9)	4 (5.9)	0.958

^a^ Data are presented as mean ± SD, median (range), or number (%), where appropriate. FS—full sternotomy, MiniAVR—aortic valve replacement via upper ministernotomy, TIA—transitory ischemic attack, CVVHD—continuous veno-venous hemodialysis, AV—atrio-ventricular, IABP—intra-aortic balloon pump, ECMO—extracorporeal membrane oxygenation, ICU—intensive care unit, EF—ejection fraction.

## Data Availability

Data supporting the reported results can be provided by the first author upon request.
